# Effect of Foot-and-Mouth Disease Virus Infection on the Frequency, Phenotype and Function of Circulating Dendritic Cells in Cattle

**DOI:** 10.1371/journal.pone.0152192

**Published:** 2016-03-23

**Authors:** Janet J. Sei, Ryan A. Waters, Mary Kenney, John W. Barlow, William T. Golde

**Affiliations:** 1 Plum Island Animal Disease Center, Agricultural Research Service, USDA, Greenport, NY, United States of America; 2 Department of Animal and Veterinary Sciences, University of Vermont, Burlington, VT, United States of America; Institut National de la Santé et de la Recherche Médicale (INSERM), FRANCE

## Abstract

Foot-and-mouth disease virus (FMDV) is a highly contagious virus that causes one of the most devastating diseases in cloven-hoofed animals. Disease symptoms develop within 2 to 3 days of exposure and include fever and vesicular lesions on the tongue and hooves. Dendritic cells (DC) play an essential role in protective immune responses against pathogens. Therefore, investigating their role during FMDV infection would lead to a better understanding of host-pathogen interactions. In this study, following infection of cattle with FMDV, we investigated the frequency and function of conventional (cDC) and plasmacytoid DC (pDC) in blood by using multi-color flow cytometry. We show that the frequency of cDC and pDC increased following FMDV infection and peaked 3 to 4 days post-infection. During peak viremia, the cattle became lymphopenic, the expression of MHC class II molecules on cDC and pDC was dramatically down-regulated, the processing of exogenous antigen by cDC and pDC was impaired, and there was an increase in IL-10 production by DC and monocytes. Notably, after clearance of FMDV from the blood, MHC class II expression returned to pre-infection levels. Altogether, our study demonstrates that in cattle, FMDV inhibits the function of DC, thereby retarding the initiation of adaptive immune responses, potentially enhancing virus shedding during the acute phase of infection.

## Introduction

Foot-and-mouth disease virus (FMDV) is a highly contagious picornavirus that causes foot-and-mouth disease (FMD) in cloven hooved animals including ruminants andswine. Clinical symptoms of FMD include pyrexia, lameness, lethargy, and development of vesicles on the feet and mouth [1]. Due to the large economical losses it causes, FMD is included in the Office International des Épizooties (OIE) list A diseases, demonstrating that it is one of the most important livestock diseases worldwide. While incubation periods in the field can be up to 14 days, in controlled experimental settings, susceptible hosts exhibit peak viremia at 1–2 days post-infection, during which time they begin developing clinical disease [1–4]. As early as 3 to 4 days post-infection, virus specific antibody is observed [5,6]. This occurs concurrently with the induction of lymphopenia in the peripheral blood by FMDV [2,4,7].

Given that dendritic cells (DC) are key for the induction of protective immune responses [8–10], understanding how DC populations function during acute FMDV infection is crucial for characterizing host-pathogen interactions. Multiple studies have demonstrated that FMDV infection in swine not only leads to a loss of peripheral blood plasmacytoid dendritic cells (pDC), but also inhibits the production of type I IFN by blood pDC [3,11]. Porcine Langerhans cells, which constitutively express IFNα, have been shown to release the cytokine upon *in vitro* exposure to FMDV [12]. However, Langerhans cells and monocyte-derived DC (moDC) isolated from FMDV infected pigs lose their ability to secrete IFNα [13]. FMDV has been reported to inhibit the maturation of *in vitro* generated porcine moDC, which affected their ability to prime T cells [14]. Moreover, a down-regulation of MHC class I expression on cells infected with FMDV *in vitro* [15] and diminished levels of MHC II molecules on murine moDC [16] have also been reported. Altogether, these findings, mostly in swine, demonstrate that FMDV inhibits the initiation of adaptive immune responses, allowing the virus to spread and subsequently shed into the environment.

We [17] and others [18] have recently characterized *ex vivo* bovine blood DC subsets by using polychromatic flow cytometry. The effect of FMDV infection on un-manipulated *ex vivo* peripheral blood cDC and pDC in cattle has not been reported. Therefore, our goal for this study was to determine whether FMDV alters frequency, expression of MHC class II molecules, cytokine production, and antigen processing of blood DC subpopulations during active infection. We surveyed four distinct bovine peripheral blood DC subsets, and monocytes following FMDV infection. We report that the frequency of the DC subsets and CD14^+^ monocytes increased during FMDV infection, and peaked at day 3 to 4 post-infection. During peak viremia, cattle peripheral blood became lymphopenic, while the expression of MHC class II on DC and CD14^+^ monocytes was dramatically down-regulated, and IL-10 production was detected in both DC and monocytes. Notably, MHC class II expression returned to pre-infection levels at 4 days post-infection, which coincided with clearance of virus from blood. Lastly, during peak viremia, FMDV inhibited the ability of DC to process exogenous antigen. These observations demonstrate that although FMDV stimulates an increase in DC and monocyte frequencies, FMDV suppresses the initiation of an effective adaptive immune response by stimulating the production of IL-10 by DC and monocytes, decreasing MHC class II expression, and inhibiting antigen processing by DC.

## Results

### Lymphopenia observed in cattle following FMDV infection

**Fig 1:**
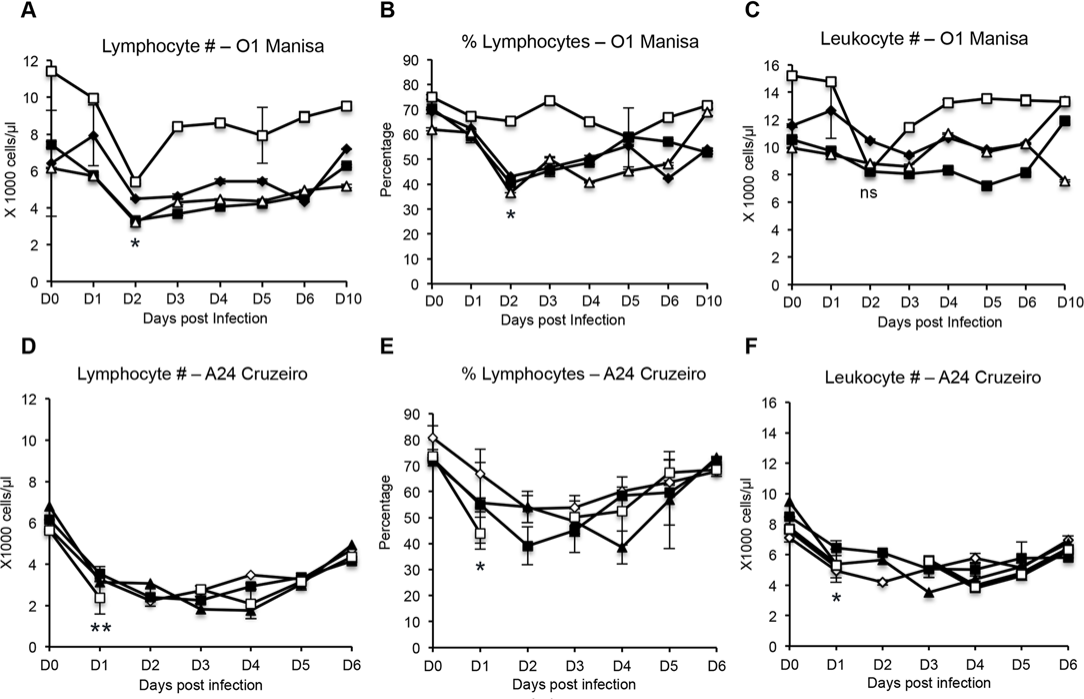
Lymphocyte and leukocyte counts following FMDV O1 Manisa and A24 Cruzeiro infection. Whole bovine blood was obtained and directly analyzed using a Hemavet 950FS analyzer to calculate absolute leukocyte numbers, and numbers or percentage of lymphocytes. Triplicate blood samples were analyzed. Each line plot depicts a single animal and error bars represent standard deviation between triplicate samples from that animal. n = 4, not significant (ns) p ≥ 0.05, * p ≤ 0.05, ** p ≤ 0.01.

Previous studies have reported that following FMDV infection of both cattle and swine, lymphopenia occurs [2,4,7]. However, conflicting studies have also reported that no lymphopenia was observed in cattle infected with FMDV [19,20]. Therefore, we investigated whether lymphopenia could be detected in cattle following infection with FMDV strain O1 Manisa or strain A24 Cruzeiro. At 2 days post- infection with FMDV O1 Manisa, we observed statistically significant decreases in lymphocyte counts (Fig 1A) and percentages (Fig 1B), however, the leukocyte numbers did not demonstrate a statistically significant difference (Fig 1C). By 10 days post-infection, the levels of lymphocytes had recovered to baseline values. Following FMDV A24 Cruzeiro infection, statistically significant decreases in lymphocyte counts, percentages and leukocyte numbers was observed at 1 day post-infection (Fig 1D and 1E). The values recovered to baseline levels by day 6. Altogether this indicated that lymphopenia is induced following FMDV infection and this lymphopenia was not serotype-specific.

### Increase in frequency of bovine peripheral blood DC subsets and monocytes following FMDV O1 Manisa Infection

**Fig 2:**
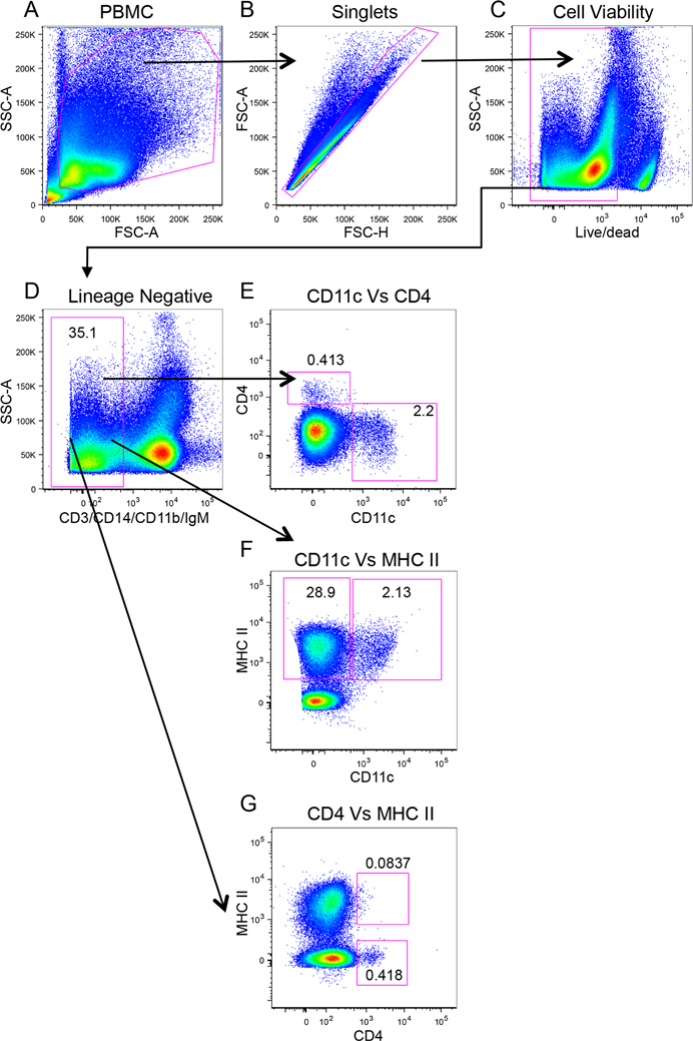
Gating strategy for the identification of bovine peripheral blood DC subsets. Five-color flow cytometry was performed to identify DC subsets in PBMC. Determination of gates was based on fluorescence minus one (FMO) controls for directly labeled antibodies and no first antibody controls when fluorescent secondary antibodies were utilized. Numbers represent percentage of cells in from parent population.

Previously, we reported the characterization of three bovine peripheral blood DC subsets: CD4^+^ pDC, CD11c^+^ cDC, and CD11c^−^ cDC [17]. As no study has assessed peripheral blood DC subsets during acute infection of cattle with FMDV, we sought to examine whether we could observe a change in their frequency during acute FMDV infection. In Fig 2, we illustrate the gating strategy to phenotypically identify blood DC subsets. From the total PBMC population (Fig 2A), doublets (Fig 2B) and dead cells (Fig 2C) were excluded. Additionally, to definitively identify DC, we excluded T cells (CD3^+^), monocytes (CD14^+^), B cells (surface IgM^+^), and CD11b^+^ cells that include CD14^−^ monocytes, NK cells, and B cells (Fig 2D) from our analyses. The lineage negative population was further assessed for expression of CD11c and CD4 (Fig 2E), which are surface molecules expressed by cDC and pDC, respectively. We observed that the expression of CD11c and CD4 was mutually exclusive, i.e. CD4^+^ DC were CD11c^−^ (0.413%), and CD11c^+^ DC were CD4^−^ (2.2%, Fig 2E). We then assessed expression of MHC class II on both CD11c (Fig 2F) and CD4 (Fig 2G) expressing cells. As we had previously described [17], 28.9% of lineage negative cells were CD11c^−^MHC class II^+^ cDC (Fig 2F), 2.13% were CD11c^+^MHC class II^+^ cDC (Fig 2F), and 0.418% were CD4^+^MHC class II^−^ pDC (Fig 2G). Notably, the proportion of CD11c^+^MHC class II^+^ cDC was similar to CD11c^+^CD4^−^ cDC (2.13% and 2.2%, respectively), and the proportion of CD4^+^CD11c^−^ pDC population corresponded with the CD4^+^MHC class II^−^ pDC (0.413% and 0.418%, respectively). This demonstrated that phenotypically, cDC were CD11c^+^MHC class II^+^CD4^−^ and pDC were CD4^+^CD11c^−^MHC class II^−^. In naïve cattle, a minor population of CD4^+^ pDC expressed MHC class II (Fig 2G, 0.0837%). However, since pathogen associated molecular patterns have been shown to up-regulate MHC class II expression on bovine CD4^+^ pDC [17], we gated on the CD4^+^ MHC class II^+^ population to determine whether we could observe an increase in the frequency of this population during FMDV infection. Hereafter, we examined the effect of acute FMDV infection on four peripheral blood DC subsets: CD11c^+^ cDC, CD11c^−^ cDC, CD4^+^MHC class II^+^ pDC, and CD4^+^MHC class II^−^ pDC, and we also included CD14^+^ monocytes.

**Fig 3:**
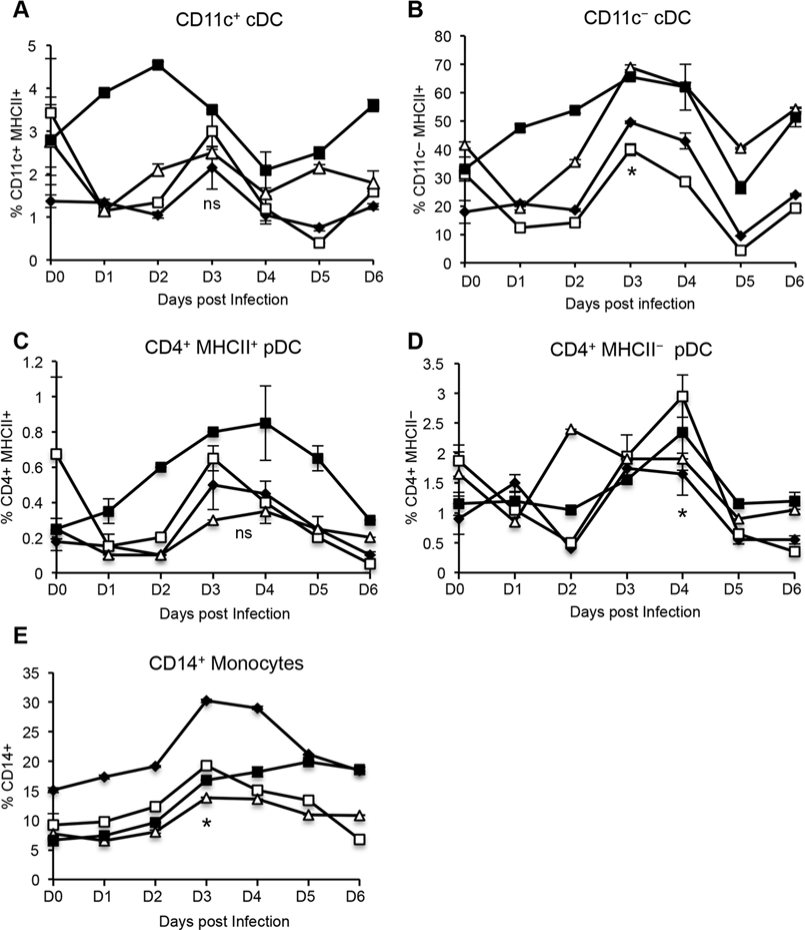
Frequency of bovine peripheral blood DC subsets and monocytes following FMDV O1 Manisa infection. Four cattle were inoculated with FMDV O1 Manisa. PBMC were isolated daily, including two baseline time points prior to infection, which were averaged. Five–color flow cytometry was performed to identify DC subsets (*A–D*) and monocyte (*E*) populations and 1 × 10^6^ events per sample were analyzed, in duplicate. Error bars represents standard deviation. Data shown represents one of two independent experiments that yielded similar results. n = 4, not significant (ns) p ≥ 0.05, * p ≤ 0.05, ** p ≤ 0.01.

Upon infection with FMDV strain O1 Manisa, there was a marked increase in the four DC subsets and CD14^+^ monocytes, with a peak in cell frequency detected between day 3 and 4 post-infection. Although a 2-fold increase in the frequency of CD11c^+^ cDC (Fig 3A) and a 3-fold increase in CD4^+^MHC class II^+^ pDC (Fig 3C) was observed, these changes were not statistically different to the levels detected prior to infection. A statistically significant 2-fold increase in CD11c^−^ cDC (Fig 3B), CD14^+^ monocytes (Fig 3E), and 3-fold increase in the frequency of and CD4^+^MHC class II^−^ pDC (Fig 3D) was observed after infection. Thus, FMDV infection caused an increase in the proportion of blood cDC, pDC and monocytes, which may reflect an influx of these cells from the bone marrow into the site of infection or secondary lymphoid organs via blood.

### Down-regulation of MHC class II molecule expression by bovine peripheral DC subsets and monocytes following FMDV O1 Manisa

**Fig 4:**
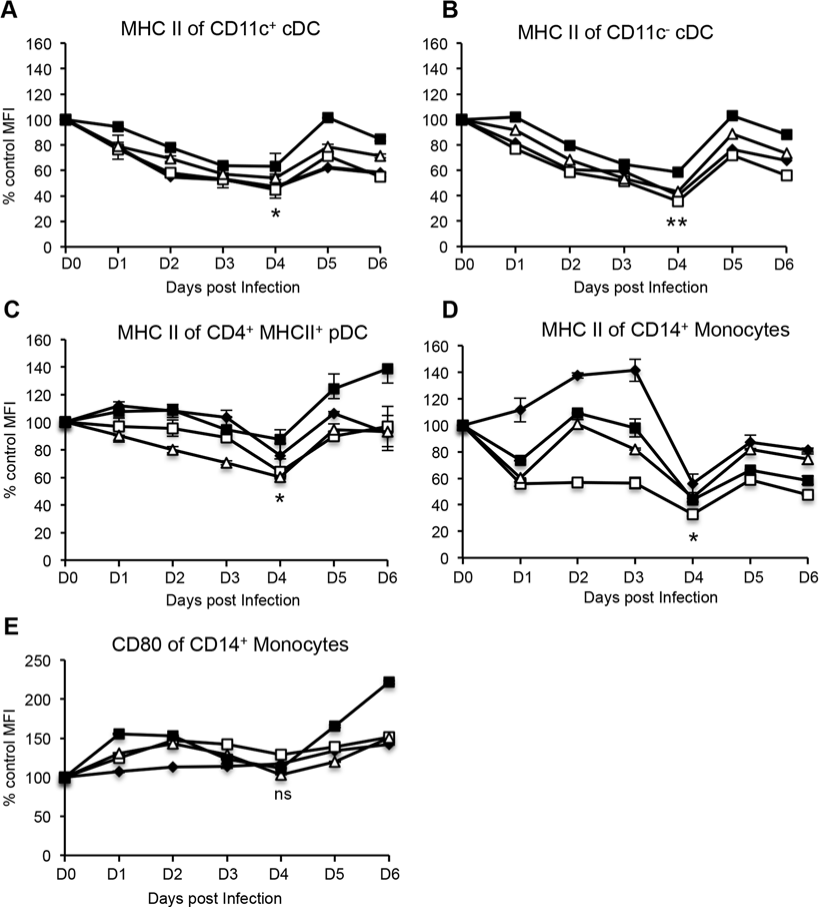
Expression of co-stimulatory molecules by bovine blood DC subsets and monocytes following FMDV O1 Manisa. Mean fluorescence intensity (MFI) of MHC class II by DC and monocytes, and MFI of CD80 by monocytes. Percent change in MHC class II and CD80 expression was determined relative to baseline values obtained prior to infection, which were normalized to 100%. Data shows one representative experiment of two independent experiments that yielded similar results. Each line plot depicts a single animal and error bars represent standard deviation between duplicate samples from that animal. n = 4, not significant (ns) p ≥ 0.05, * p ≤ 0.05, ** p ≤ 0.01.

Previous studies have reported that *in vitro* infection of murine moDC [16] and porcine moDC [14] with FMDV O1 Campos and FMDV C-S8c1, respectively, down-regulates the expression of MHC class II molecules. Since professional antigen presenting cells (pAPC) activate naïve CD4^+^ T cells by presenting cognate antigen on MHC class II molecules, we sought to determine whether FMDV infection of cattle affected the expression of MHC class II molecules on blood DC subsets and monocytes during acute infection. The expression level of MHC class II molecules on the surface of the DC subsets and monocytes was determined by assessing the mean fluorescent intensity (MFI) of MHC class II. The MFI of MHC class II for these populations at baseline (prior to infection) was normalized to 100%. Any changes in MFI of MHC class II at various days post-infection, was expressed as a percentage of the baseline MFI. Following infection with FMDV O1 Manisa, we observed a down-regulation in the expression of MHC class II on the blood DC subsets. Specifically, a statistically significant ~50% decrease in the levels of MHC class II expression by CD11c^+^ cDC (Fig 4A), CD11c^−^ cDC (Fig 4B), CD4^+^ MHC class II^+^ pDC (Fig 4C), and CD14^+^ monocytes (Fig 4D) was observed at 4 days post-infection, and thereafter recovered to baseline levels. Unlike MHC class II molecules, the expression of the co-receptor ligand for T cells, CD80, was unaltered on CD14^+^ monocytes following FMDV infection (Fig 4E). Altogether, the data suggests that during acute infection, FMDV interferes with antigen presentation to T cells by down-regulation of MHC class II molecules on DC and monocytes.

### Increase in frequency of blood DC subsets & monocytes, and down-regulation of MHC class II molecules following FMDV strain A24 Cruzeiro infection

**Fig 5:**
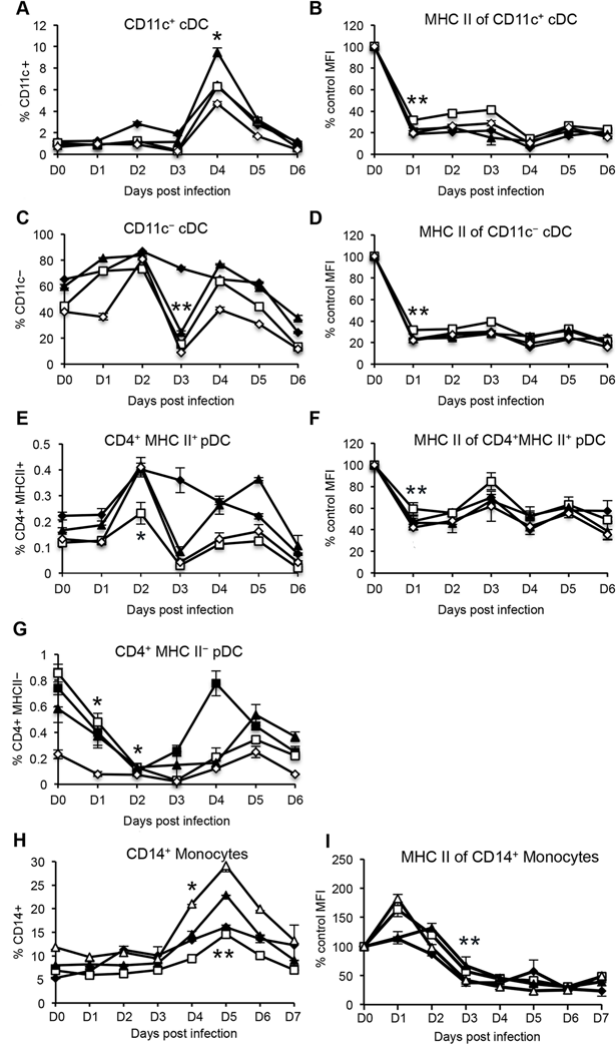
Frequency of peripheral blood DC, monocytes and MHC class II expression following FMDV A24 Cruzeiro infection. Four cattle were inoculated with FMDV A24 Cruzeiro. PBMC were isolated daily, including a baseline blood-draw prior to infection. Five–color flow cytometry was performed to identify DC and CD14^+^ monocyte populations. Expression of MHC class II molecules was determined by quantifying MFI values as described in Fig 3. Each line plot depicts a single animal and error bars represent standard deviation between duplicate samples from that animal. n = 4, not significant (ns) p ≥ 0.05, * p ≤ 0.05, ** p ≤ 0.01.

To determine whether the observations demonstrating an increase in DC and monocyte frequency (Fig 3), and down-regulation of MHC class II molecules (Fig 4) were FMDV O1 Manisa specific, we performed similar analyses on FMDV A24 Cruzeiro infected cattle. We found a statistically significant 5-fold increase in the frequency of CD11c^+^ cDC observed at 4 days post-infection (Fig 5A), and a statistically significant 80% decline in the levels of MHC class II observed at 1 day post-infection, which then remained low throughout the first week after FMDV infection (Fig 5B). There was a statistically significant decrease in the frequency of CD11c^−^ cDC observed at day 3 post infection (Fig 5C), and a statistically significant 80% decline in the levels of MHC class II by CD11c^−^ cDC beginning at 1 day post-infection (Fig 5D). A statistically significant 2-fold increase in the proportion of CD4^+^MHC class II^+^ pDC (Fig 5E) observed at 2 days post-infection, the cell numbers rapidly declined at day 3 post-infection returning to pre-infection levels. Similarly, there was a statistically significant ~50% decrease in expression of MHC class II molecules by CD4^+^MHC class II^+^ pDC (Fig 5F). We observed a statistically significant decline in the frequency of CD4^+^MHC class II^−^ pDC at 1 and 2 days post-infection, then the levels recovered to baseline thereafter (Fig 5G). A statistically significant 3-fold increase in the percentage of CD14^+^ monocytes was also observed, which peaked at 5 days post-infection (Fig 5H), and the MHC class II expression declined by approximately 70% at day 3 post-infection (Fig 5I). We also assessed the expression of CD80 by CD11c^+^ DC and CD14^+^ monocytes but did not observe any significant changes after FMDV A24 Cruzeiro infection (S1 Fig). Altogether, our results demonstrate that similar to FMDV strain O1 Manisa, infection of cattle with strain A24 Cruzeiro led to an overall increase in DC and monocyte frequency, and a decline in MHC class II molecule expression.

### Production of IL-10 by peripheral blood DC and monocytes following FMDV infection

**Fig 6:**
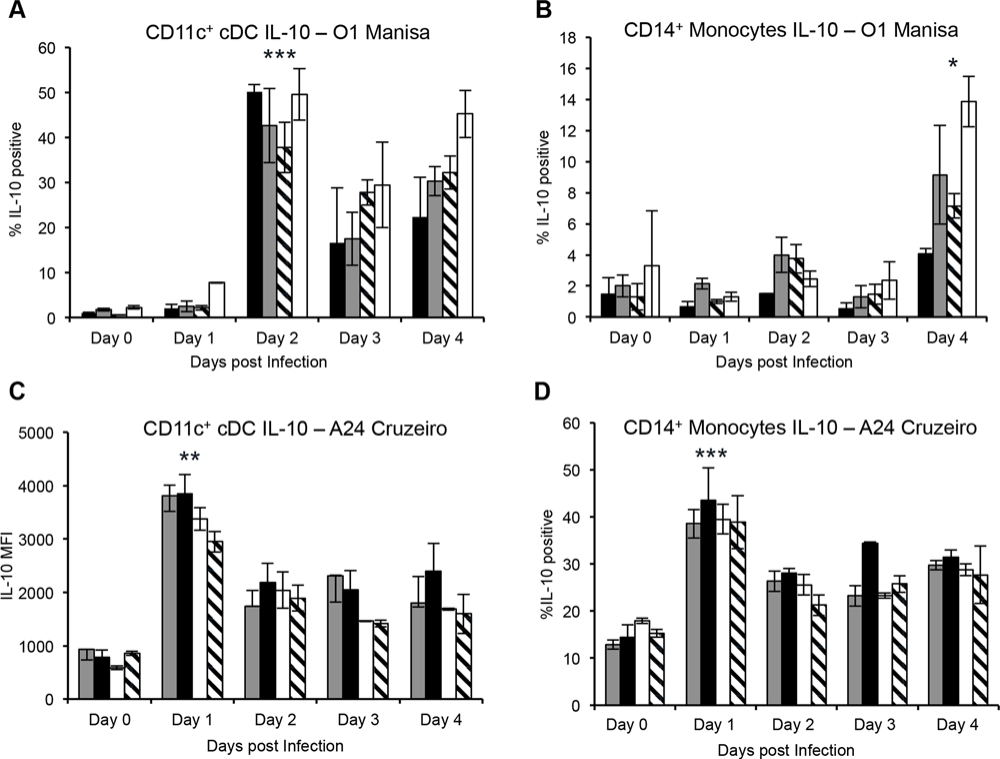
Production of IL-10 by bovine peripheral blood DC and monocytes following FMDV O1 Manisa and A24 Cruzeiro infection. Intracellular cytokine staining was performed to determine whether blood cDC and monocytes produce IL-10 following infection of cattle with FMDV O1 Manisa (*A* and *B*) or A24 Cruzeiro (*C* and *D*). Data shows one representative experiment of two independent experiments that demonstrated similar results. Each bar plot and line depicts a single animal and error bars represent standard deviation between duplicate samples from that animal. n = 4, not significant (ns) p ≥ 0.05, * p ≤ 0.05, ** p ≤ 0.01, *** p ≤ 0.001.

Previous studies have demonstrated that FMDV induced IL-10 production by porcine moDC was directly linked to down-regulation of MHC class II molecules, which led to the inhibition of T cell activation [14]. In cattle, IL-10 production has been detected in the sera of FMDV infected animals with a peak at day 3 or 4 post-infection, which coincides with the development of clinical disease [20]. Indeed, we also confirmed that upon FMDV O1 Manisa and A24 Cruzeiro (S2 Fig) infection, we could detect IL-10 in cattle sera, which peaked at day 4 following infection with strain A24, but was variable in four steers infected with O1Manisa (S2 Fig). As our studies demonstrated that MHC class II molecules on blood DC subsets and monocytes were down-regulated following FMDV infection, we sought to investigate whether the immunosuppression observed was mediated by IL-10. Peripheral blood cDC and monocytes were assessed for IL-10 expression directly *ex vivo* following FMDV O1 Manisa and A24 Cruzeiro infection. A statistically significant increase in the production of IL-10 by CD11c^+^ DC, was detected at 2 days post FMDV O1 Manisa infection, and was sustained until 4 days post-infection (Fig 6A). Similarly, a statistically significant increase in IL-10 production by CD14^+^ monocytes was detected at 4 days post FMDV O1 Manisa infection (Fig 6B). At 1 day post FMDV A24 Cruzeiro infection, a statistically significant increase in the expression of IL-10 by CD11c^+^ DC (Fig 6C) and CD14^+^ monocytes (Fig 6D) was observed. In summary, FMDV infection of cattle is characterized by production of IL-10 by DC and monocytes, which may be responsible to immunosuppression in cattle, characterized by down-regulation of MHC class II molecules and lymphopenia.

### Inhibition of antigen processing by blood DC subsets following FMDV infection

**Fig 7:**
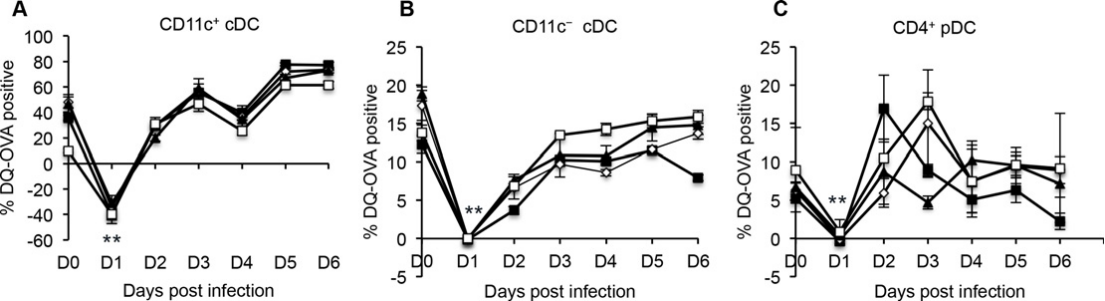
Processing of exogenous antigen by bovine blood DC subsets following FMDV A24 Cruzeiro infection. PBMC were isolated at various days following infection of cattle with FMDV A24 Cruzeiro. PBMC were then incubated with DQ-OVA to assess the antigen processing function of DC at 4°C or 37°C. For graphical analysis, control 4°C values were subtracted from samples incubated at 37°C. Each line plot depicts a single animal and error bars represent standard deviation between duplicate samples from that animal. n = 4, ** p ≤ 0.01.

Given that we had observed evidence of FMDV-mediated immunosuppression, we examined whether the antigen processing function on DC was also affected by FMDV infection. Antigen processing by DC subsets was determined by incubating the cells with a self-quench conjugate of ovalbumin (DQ-OVA) to PBMC isolated from FMDV A24 Cruzeiro infected animals. Proteolytic cleavage of DQ-OVA by DC was detected via flow cytometry. Background fluorescence of un-cleaved DQ-OVA (4°C) was subtracted from cleaved DQ-OVA (37°C) at various days following infection. A statistically significant decline in the antigen processing ability of CD11c^+^ cDC (Fig 7A), CD11c^−^ cDC (Fig 7B), and CD4^+^ pDC (Fig 7C) was observed at 1 day post-infection, and this returned back to baseline levels by day 2 to 3 after infection. This time period corresponds to peak viremia and onset of clinical disease.

## Discussion

Recent investigations by ourselves [17] and others [18] had identified novel lineage negative CD11c^+^ cDC, CD11c^−^ cDC, and CD4^+^ pDC subsets in bovine blood. During acute FMDV infection, our analysis of the DC sub-populations and monocytes in blood circulation of cattle shows an overall increase in the frequency of CD11c^+^ cDC, CD11c^−^ cDC, CD4^+^ pDC, and monocytes, and a decline in MHC class II expression within the first 3 to 4 days post FMDV infection. In other species, an increase in the frequency of blood DC has been reported during malaria [21] and simian immunodeficiency virus [22] infections, asthma [23], and intensive exercise [24]. Following pathogen stimulation, DC undergo maturation, which is characterized by increased expression of MHC class II molecules on the surface of the cells [17,25]. During FMDV, strain O1 Manisa infection of cattle, we observed a statistically significant (50%) decline in the levels of MHC class II on CD11c^+^ cDC, CD11c^−^ cDC, and CD4^+^ pDC until day 4 post-infection, when they returned to pre-infection levels. Infection of cattle with FMDV strain A24 led to a greater (~80%) reduction in MHC II expression on DC populations and in this instance, the expression never recovered during the time we conducted these analyses. This may suggest that FMDV A24 Cruzeiro is a more aggressive pathogen in that it induces a stronger immunosuppressive signal in cattle than O1 Manisa. Indeed, production of the anti-inflammatory cytokine IL-10 by CD11c^+^ cDC following FMDV A24 Cruzeiro occurred 1 day earlier than O1 Manisa, and was sustained for several days post-infection.

Given that clearance of FMDV viremia typically occurs at 4 days post-infection [2], our finding demonstrates that MHC class II down-regulation on DC correlates with the presence of FMDV in blood. While we did not determine whether the blood cDC are infected by FMDV, our finding that MHC class II expression recovered after clearance of the viremia (with O1 Manisa infection), suggests that either direct or indirect contact with FMDV may be responsible for down-regulation of MHC class II molecules. Alternatively, the decrease in MHC II on DCs could be in response to type I IFN secretion by virus-infected or stimulated stromal cells including epithelial cells, mucosal DCs, and mucosal macrophages.

IL-10 has been shown to inhibit the egress of MHC class II molecules from the MHC class II compartment to the plasma membrane, and prevent the recycling of MHC class II molecules from the plasma membrane by accumulating the molecules in intracellular vesicles [26]. Various viruses utilize IL-10 as a viral evasion mechanism including classical swine fever [27], porcine reproductive and respiratory syndrome virus [28,29], dengue [30,31], and West Nile virus [32]. We and others have demonstrated that IL-10 can be detected in sera of FMDV infected cattle [20]. We now show that CD11c^+^ cDC and monocytes are a source of IL-10 *in vivo* following infection of cattle with FMDV O1 Manisa and A24 Cruzeiro.

In addition to down-regulation of MHC class II molecule expression, FMDV A24 Cruzeiro infection of cattle led to a statistically significant reduction in the antigen processing function of CD11c^+^ cDC at 1 day post-infection (peak viremia). By dampening cDC function, FMDV affects naïve T cell activation. Certainly, we observed lymphopenia in FMDV O1 Manisa and A24 Cruziero infected cattle that coincided with the peak in viremia. Lymphopenia has previously been reported in FMDV infected swine [2,4,7] and cattle [33]. However, two studies found that neither lymphopenia nor dampening of T cell functions was observed following FMDV infection [20]. Differences between FMDV serotypes, whether virus was harvested directly from animals or adapted *in vitro*, and route of infection may account for these conflicting results. Additionally, total leukocyte counts were assessed as a measure of lymphopenia upon FMDV O UKG infection [20]. Since leukocytes include multiple cell types such as granulocytes, phagocytes and lymphocytes, a more accurate assessment of lymphopenia would be the quantification of absolute lymphocyte counts. Indeed, in agreement with Windsor et. al. findings [20], we did not see a statistically significant change in the leukocyte numbers when cattle were infected with FMDV O1 Manisa. Moreover, in studies that demonstrated FMDV-mediated lymphopenia in cattle, the frequency of lymphocytes was assessed [4], which is in agreement with our findings.

Interestingly, following infection with FMDV A24 Cruzeiro, we observed a statistically significant decline in the frequency of CD11c^−^ cDC at day 3 post-infection, then the cell numbers recovered to pre-infection levels. Our previous study characterizing blood DC subsets of cattle showed that CD11c^−^ cDC differentiate into CD11c^+^ cDC in the presence of GM-CSF and IL-4 [17]. Indeed, levels of GM-CSF have been demonstrated to increase during inflammatory responses [34]. Therefore, it is plausible that the decline in blood CD11c^−^ cDC post FMDV A24 Cruzeiro infection, may be due to the differentiation of CD11c^−^ cDC into CD11c^+^ cDC. Since cDC have a short lifespan of about 2–3 days [35–37], and considering that during infection, blood cDC migrate into secondary lymphoid organs [38] or lymphoid tissues [39], the continuous replenishment of cDC is required. Interestingly, the numbers of CD11c^+^ cDC in turn peaked at day 4 post-infection. Alternatively, the decline in CD11c^−^ cDC may be due to migration into lymph nodes, spleens or sites of FMDV inflammation where they differentiate into mature cDC.

In blood of naïve cattle, the majority of type I IFN producing CD4^+^ pDC are immature MHC class II^−^ cells [17]. We previously described the loss of porcine blood CD4^+^ pDC during peak FMDV viremia [3]. Here, we report infection of cattle with FMDV A24 Cruzeiro leads to a rapid decline in immature CD4^+^MHC class II^−^ pDC during peak viremia. We also observed a down-regulation of the antigen processing function of CD4^+^MHC class II^−^ pDC following infection with FMDV A24 Cruzeiro. This demonstrates that FMDV A24 Cruzeiro may suppress the function of immature pDC, such as type I IFN production. Indeed, in addition to loss of the cells from the periphery, we reported loss of pDC function during peak FMDV viremia in swine [3]. For mature CD4^+^MHC class II^+^ pDC, while infection with FMDV O1 Manisa and A24 Cruzeiro increased their frequency, their expression of MHC class II significantly declined during FMDV infection.

*In vitro*, blood monocytes have also been shown to differentiate to cDC in the presence of exogenous GM-CSF and IL-4 [40–44]. We therefore sought to determine their frequency following FMDV infection. In this study, we report that CD14^+^ monocyte frequency increases following inoculation of cattle with both FMDV O1 Manisa and A24 Cruzeiro. These results are in agreement with a previous report in cattle which demonstrated that blood monocyte numbers increased following vaccination and challenge with FMDV [45]. An increase in blood monocytes may function as a reservoir for cDC. Similar to cDC, the levels of MHC class II declined and IL-10 was detected in monocytes following FMDV infection. This suggests that FMDV may also affect antigen presentation function of monocytes.

In conclusion, results presented here show that FMDV infection in cattle induces a transient suppression of immune function that corresponds to peak viremia. Acute FMDV was characterized by lymphopenia, and a fold-increase increase in the frequency of blood cDC, pDC, and monocyte populations. We also observed a down-regulation of MHC class II molecule expression and an inhibition of antigen processing function by cDC and pDC. This dampening of APC function may be caused by FMDV-mediated IL-10 production, which we also detected during peak viremia. These data demonstrate a mechanism by which FMDV inhibits or delays naïve T cell activation by transiently suppressing DC function during acute infection.

## Materials and Methods

### Ethics Statement

The Plum Island Animal Disease Center, Institutional Animal Care and Use Committee, designated PIACUC, operates under policies published in the “Guide”, Animal Welfare Regulations and PHS Policy, Public Health Service (PHS) of the US Department of Health and Human Services (HHS). The animal use protocol 219-11-R, Dr. W.T. Golde, principal investigator, was approved by the committee for the period of June 1, 2011 to May 31, 2014. All experiments reported here were carried out under this protocol at the Plum Island Animal Disease Center.

### Animals

All experiments were performed in a secure biosafety level three laboratory at Plum Island Animal Disease Center (PIADC). Holstein cattle, 4–6 months of age that weighed 400 pounds, were transferred from the Miller Research Barn at the University of Vermont (Burlington, VT) or were purchased from Thomas Morris (Springfield, PA). Animals were acclimated for at least 1 week before experimentation. Four cattle were investigated in each group.

### Viruses

FMDV strains O1 Manisa and A24 Cruzeiro were investigated in this study. As previously described, both FMDV viruses were isolated from the tongue epithelium macerate harvested from two cattle infected with FMDV [46]. Virus aliquots were maintained and stored at −70°C until use. The challenge virus was titrated in the tongue of a cow to determine 50% bovine tongue infectious doses (BTID_50_). Cattle were sedated and inoculated intradermal lingually with 1 × 10^4^ BTID_50_ in four regions of base the tongue. Animals were examined daily for clinical signs such as fever, lameness, lethargy, and development of vesicles on the feet and in the mouth. All animals developed fever on days 1 to 3 following infection that resolved in 48 hours after onset. Following FMDV infection, all cattle had vesicles on all four feet when examined on day 4 and these lesions were beginning to resolve on clinical inspection 7 days after inoculation.

### Isolation of PBMC

Peripheral blood mononuclear cells (PBMC) were isolated via leukophoresis as previously described [17]. PBMC were resuspended in RPMI-1640 (Life Technologies, Grand Island, NY), supplemented with 10% heat-inactivated FBS (Thermo Scientific, Waltham, MA), 1X L-glutamine (Life Technologies), 1X anti-biotic/anti-mycotic (Life Technologies), and 50 μM of 2-Mercaptoethanol (Sigma-Aldrich, St. Louis, MO). Alternatively, PBMC were isolated following red blood cell (RBC) lysis with ACK Lysing buffer (Quality Biological Inc, Gaithersburg, MD). Whole blood was incubated at room temperature for 10 minutes and cells were washed twice in PBS then resuspended in supplemented RPMI-1640. To determine blood cell counts, samples were analyzed using a Hemavet 950 analyzer (Drew Scientific,Waterbury, CT).

### Cell surface marker staining

PBMC were aliquoted into plates at 1 × 10^6^ cells per well and washed once with 1X PBS. For cell viability, LIVE/DEAD® Fixable Yellow Dead Cell Stain Kit (Life Technologies, Grand Island, NY) or 7-Aminoactinomycin D (7-AAD) (BD Biosciences, San Jose, California) were used. When using LIVE/DEAD® Fixable Yellow Dead Cell Stain Kit, cells were stained with the dye for 30 minutes at room temperature prior to staining cells with antibodies. 7-AAD staining was performed following staining of surface markers with antibodies. The following primary anti-bovine antibodies were used: anti-CD11c (IgM, BAQ153A; Washington State University (WSU), Pullman, WA), anti-CD3 (IgG1, MM1A; WSU), anti-CD14 (IgG1, MM61A; WSU), anti-CD11b (IgG1, MM12A; WSU), anti-IgM (IgG1, IL-A30; AbD Serotec, Raleigh, NC), anti-MHC class II R-PE (IgG2a, IL-A21; AbD Serotec), and anti-CD4 Alexa 647 (IgG2a, CC8; AbD Serotec). The secondary antibodies used were rat anti-mouse IgM PE-Cy7 (II/41; eBiosciences, San Diego, CA), rat anti-mouse IgG1 BV421 (A85-1;BD Biosciences, San Jose, California). Antibodies were diluted in FACS buffer (0.3% BSA, 0.9% sodium azide, PBS) and staining was conducted on ice for 20 minutes. Analysis was performed using an LSR-II (BD Biosciences, San Jose, California), FACS DIVA Software (BD Biosciences, San Jose, California), and FlowJo Software 9.6.4 Version (TreeStar, Ashland, USA).

### Intracellular cytokine staining

One million PBMC in supplemented RPMI-1640 were incubated with 1X final concentration of Brefeldin A (eBiosciences, San Diego, CA) for 5 hours at 37°C, 5% CO_2_. Cells were washed once with PBS then stained with LIVE/DEAD® Fixable Yellow Dead Cell Stain for 30 minutes. The PBMC were stained with the following primary antibodies for another 30 minutes: anti-human CD14-APC (TÜK4; Miltenyi Biotech Inc, San Diego, CA), anti-bovine CD11b (IgG2b, MM10A; AbD Serotec, Raleigh, NC), anti-bovine CD11c (IgM, BAQ153A; WSU, Pullman, WA). The following secondary antibodies were incubated with the cells for another 30 minutes: goat ant-mouse IgG2b PE-Cy7 (Southern Biotech, Birmingham, Alabama), goat ant-mouse IgM FITC (Southern Biotech, Birmingham, Alabama). Fixation with 1% paraformaldehyde was performed for 10 minutes at room temperature, and cells were placed at 4°C overnight in FACS buffer. The next day, PBMC were permeabilized with 1X BD FACS Perm II Buffer (BD Biosciences, San Jose, California) at room temperature for 10 minutes, and stained with anti-IL-10 biotin (IgG1, CC320; AbD Serotec) for 20 minutes on ice. To detect the biotinylated IL-10, cells were stained with BV421 Streptavidin (Biolegend, San Diego, CA) for 20 minutes. Acquisition and analysis was performed on a BD LSR-II using the BD DIVA software.

### DQ-OVA Assay

Stimulation and surface marker staining were performed as previously described [17]. Acquisition was performed on a BD LSR-II using the BD DIVA software and analysis was done using Flowjo Software 9.6.4 Version (TreeStar, Ashland, USA).

### Detection of IL-10 in serum by ELISA

At various days post FMDV O1 Manisa and FMDV A24 Cruzeiro infection, serum was collected and stored at -70°C until assay. The production of IL-10 was determined by using a commercial solid-phase competitive ELISA kit (anti-Interleukin 10 BioAssay^TM^ ELISA Kit (bovine) from United States Biological (Salem, MA). As recommended by the manufacturer, each sample was run in duplicate and the average OD values were interpolated from a four-parameter logistic curve to determine the IL-10 concentration in each sample.

### Statistical Analysis

Two-tailed paired Student’s t-test was performed using the Prism software (GraphPad, La Jolla, CA). Significant differences were identified using p-values: not significant (ns) p ≥ 0.05, * p ≤ 0.05, ** p ≤ 0.01, *** p ≤ 0.001, **** p ≤ 0.0001.

## Supporting Information

S1 Fig Additional experiments showing CD80 expression, frequency, MHC class II expression, and IL-10 production by CD11c^+^ DC & CD14^+^ monocytes upon FMDV O1 Manisa and A24 Cruzeiro infection. (TIF)

S2 Fig Serum levels of IL-10 in μg/ml from FMDV O1 Manisa and A24 Cruzeiro infected cattle. (TIFF)
