# Supplementary figures and images for: Effect of Foot-and-Mouth Disease Virus Infection on the Frequency, Phenotype and Function of Circulating Dendritic Cells in Cattle
Source: PLoS One. 2016 Mar 23;11(3):e0152192. doi: 10.1371/journal.pone.0152192 (PMC4805171; doi:10.1371/journal.pone.0152192)

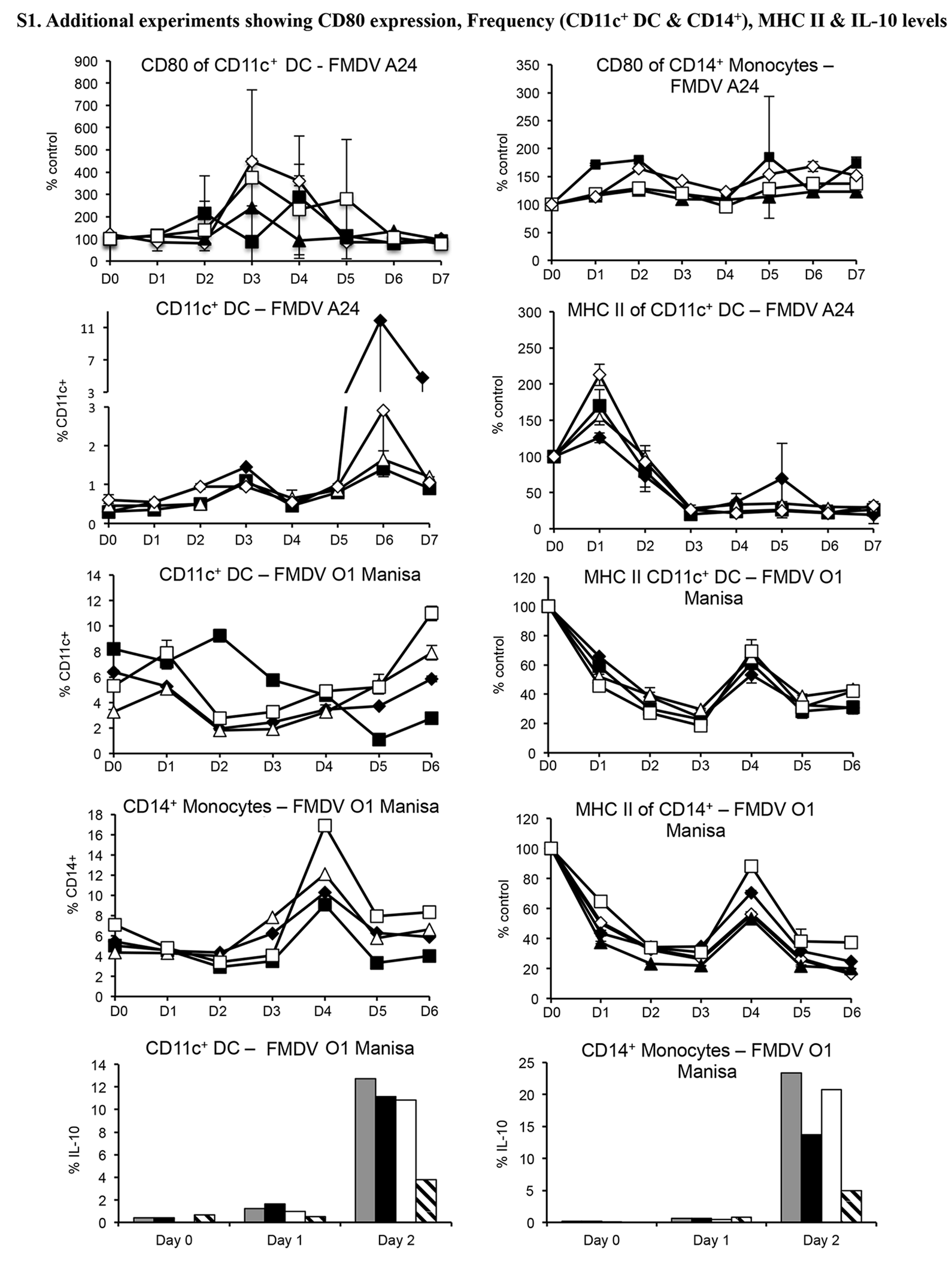

Supplement: S1 Fig — (TIF) [file pone.0152192.s001.tif]

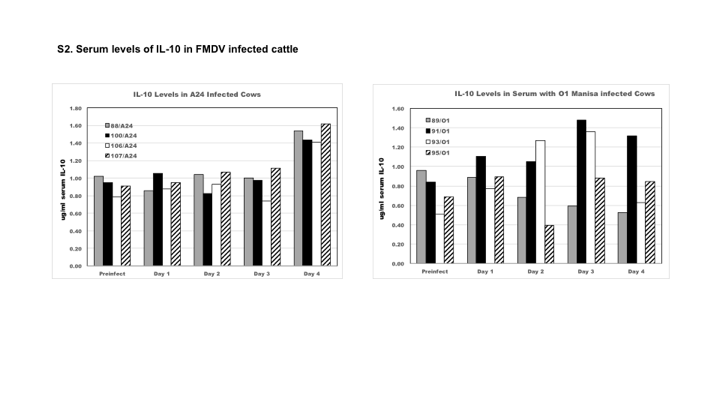

Supplement: S2 Fig — (TIFF) [file pone.0152192.s002.tiff]
